# Associative Learning of Stimuli Paired and Unpaired With Reinforcement: Evaluating Evidence From Maggots, Flies, Bees, and Rats

**DOI:** 10.3389/fpsyg.2018.01494

**Published:** 2018-08-24

**Authors:** Michael Schleyer, Markus Fendt, Sarah Schuller, Bertram Gerber

**Affiliations:** ^1^Department Genetics of Learning and Memory, Leibniz Institute for Neurobiology, Magdeburg, Germany; ^2^Institute for Pharmacology and Toxicology, Otto von Guericke University Magdeburg, Magdeburg, Germany; ^3^Center for Behavioral Brain Sciences, Magdeburg, Germany; ^4^Behavior Genetics, Institute for Biology, Otto von Guericke University Magdeburg, Magdeburg, Germany

**Keywords:** safety learning, fear conditioning, reward, punishment, memory valence

## Abstract

Finding rewards and avoiding punishments are powerful goals of behavior. To maximize reward and minimize punishment, it is beneficial to learn about the stimuli that predict their occurrence, and decades of research have provided insight into the brain processes underlying such associative reinforcement learning. In addition, it is well known in experimental psychology, yet often unacknowledged in neighboring scientific disciplines, that subjects also learn about the stimuli that predict the absence of reinforcement. Here we evaluate evidence for both these learning processes. We focus on two study cases that both provide a baseline level of behavior against which the effects of associative learning can be assessed. Firstly, we report pertinent evidence from *Drosophila* larvae. A re-analysis of the literature reveals that through paired presentations of an odor A and a sugar reward (A+) the animals learn that the reward can be found where the odor is, and therefore show an above-baseline preference for the odor. In contrast, through unpaired training (A/+) the animals learn that the reward can be found precisely where the odor is not, and accordingly these larvae show a below-baseline preference for it (the same is the case, with inverted signs, for learning through taste punishment). In addition, we present previously unpublished data demonstrating that also during a two-odor, differential conditioning protocol (A+/B) both these learning processes take place in larvae, i.e., learning about both the rewarded stimulus A and the non-rewarded stimulus B (again, this is likewise the case for differential conditioning with taste punishment). Secondly, after briefly discussing published evidence from adult *Drosophila*, honeybees, and rats, we report an unpublished data set showing that relative to baseline behavior after truly random presentations of a visual stimulus A and punishment, rats exhibit memories of opposite valence upon paired and unpaired training. Collectively, the evidence conforms to classical findings in experimental psychology and suggests that across species animals associatively learn both through paired and through unpaired presentations of stimuli with reinforcement – with opposite valence. While the brain mechanisms of unpaired learning for the most part still need to be uncovered, the immediate implication is that using unpaired procedures as a mnemonically neutral control for associative reinforcement learning may be leading analyses astray.

## Introduction

Finding rewards and avoiding punishments are powerful goals of behavior in insects and vertebrates, including humans. To maximize rewards, for example, it is beneficial to learn about the stimuli that predict where and when they can be found. However, it can be equally important to learn where and when a reward will not be found (Rescorla, 1967; Malaka, 1999). Although well established in the classical experimental psychology literature, the latter learning process is frequently left out of consideration even in immediately neighboring fields of study. This can be problematic because research into the brain mechanisms of learning and memory, for example, may go astray if it fails to take into account both of these processes when designing control procedures for the effects of associative learning. Here we focus non-exclusively on two cases of Pavlovian conditioning, one in larval *Drosophila* and the other in rats, which provide different types of control procedure for determining a baseline behavior against which the effects of learning both through reinforcement and non-reinforcement can be assessed.

In Pavlovian conditioning, a stimulus A (in Pavlovian terminology: the conditioned stimulus or CS) is presented along with a reinforcer + (in Pavlovian terminology: the unconditioned stimulus or US). By such paired A+ training, an association is formed between A and the reinforcer (Pavlov, 1927). In the past few decades, powerful theories have been introduced to explain such reinforcement learning. Many of them feature what is known as the delta rule (Rescorla and Wagner, 1972; Mackintosh, 1975; Rumelhart et al., 1986; Van Hamme and Wasserman, 1994; Malaka, 1999) (**Supplementary Figure S1A**). Essentially, this rule holds that the more we remember, the less we learn. In other words, the amount of reward learning about A depends on the difference between the reward received in the presence of A minus the reward predicted by A, the so-called ‘prediction error’. Considering multiple training trials (A+, A+, A+, etc.), the prediction error is large and positive for the first A+ trial. This is because much more reward is received than is predicted (‘pleasant surprise’). As training progresses, the reward will eventually be fully predicted such that the prediction error is zero and no further learning accrues to A.

Already in early studies in the field, unpaired training was introduced as a control procedure for reinforcement learning (e.g., Harris, 1943). In such a procedure, A and the reinforcer never occur in temporal proximity (A/+ for the case of reward learning, A/- for the case of punishment learning). Later, however, Rescorla (1966, 1967, 1968) demonstrated that animals can learn through such unpaired presentations: specifically, they can learn that A predicts the absence of the reinforcer. How is this possible? Doesn’t it violate the principles of association to suggest that a stimulus A presented without reinforcement is learned about? And if A is presented unpaired from reward, for example, how is it possible that A comes to predict where reward is not, rather than where punishment, or the spaghetti monster, is not? In fact, delta-rule types of model for reinforcement learning can offer an explanation. The assumption is that during for example a reward-only trial (+), an association is formed between the experimental context and the reward (Dweck and Wagner, 1970; Rescorla, 1972; Grau and Rescorla, 1984; Bouton and Nelson, 1998) (‘context’ being understood as the totality of stimuli that are not manipulated during the experiment). When in a subsequent trial A is presented within the same context, this context-reward association will predict the reward. As the reward is not actually present, however, a negative prediction error arises: less reward is received than is contextually predicted (‘unpleasant surprise’). This negative prediction error will then be associated with A, which in consequence becomes a signal for no-reward (rather than remaining neutral, i.e., not being a signal for anything) (Rescorla, 1966, 1967, 1968). Thus, as a result of A+ training, A signals where the reward can be found, whereas after A/+ training A signals where the reward cannot be found. Most of the remainder of the present paper is about strategies for studying A+ and A/+ learning, and about the implications of these learning processes for designing control procedures for reinforcement learning. We first review the literature on larval *Drosophila* that provides evidence for A+ and A/+ learning relative to a control condition that prevents the behavioral expression of associative memories. Then we report on so far unpublished experiments regarding these learning processes in differential conditioning in this paradigm, and briefly evaluate pertinent literature on adult flies, honey bees and rats. Finally, we present unpublished data demonstrating associative learning through paired and unpaired training in a fear-conditioning paradigm in rats, relative to a control condition that prevents the formation of associative memories.

## Unpaired-Memory in Larval *Drosophila*?

Odor-taste associative learning in the *Drosophila* larva is an ecologically plausible study case for Pavlovian conditioning (reviews include Gerber and Stocker, 2007; Diegelmann et al., 2013; Widmann et al., 2017; see also Aceves-Pina and Quinn, 1979 for a pioneering approach using odor-electric shock learning). In Pavlovian terminology, the odor would be designated the CS, and the tastant the US. The rich toolbox for transgenic manipulation available for *Drosophila*, the numerically simple larval brain consisting of only about 10,000 neurons, and the upcoming cellular atlas and synaptic connectome of its nervous system allow for experiments with enticing analytical resolution (Venken et al., 2011; Li et al., 2014; Eichler et al., 2017). Despite the simplicity of their brains, larvae learn to associate odor stimuli with taste rewards such as sugar, or with bitter tastants such as quinine as a punishment (Scherer et al., 2003; Gerber and Hendel, 2006). They further show discrimination, generalization, memory consolidation, and an organization of learned behavior according to its expected outcome (Gerber and Hendel, 2006; Mishra et al., 2010; Chen et al., 2011; Schleyer et al., 2011; Chen and Gerber, 2014; Schleyer et al., 2015a,b; Widmann et al., 2016). Last but not least, the transparent cuticle of larvae allowed for the first use of Channelrhodopsin-2 to remote-control central brain neurons in a behaving animal (Schroll et al., 2006). Thus, the larva is simple enough to be studied with ease and precision, and complex enough for this to be worth the effort.

### How to Determine Baseline Odor Preferences in Larval *Drosophila*

**FIGURE 1:**
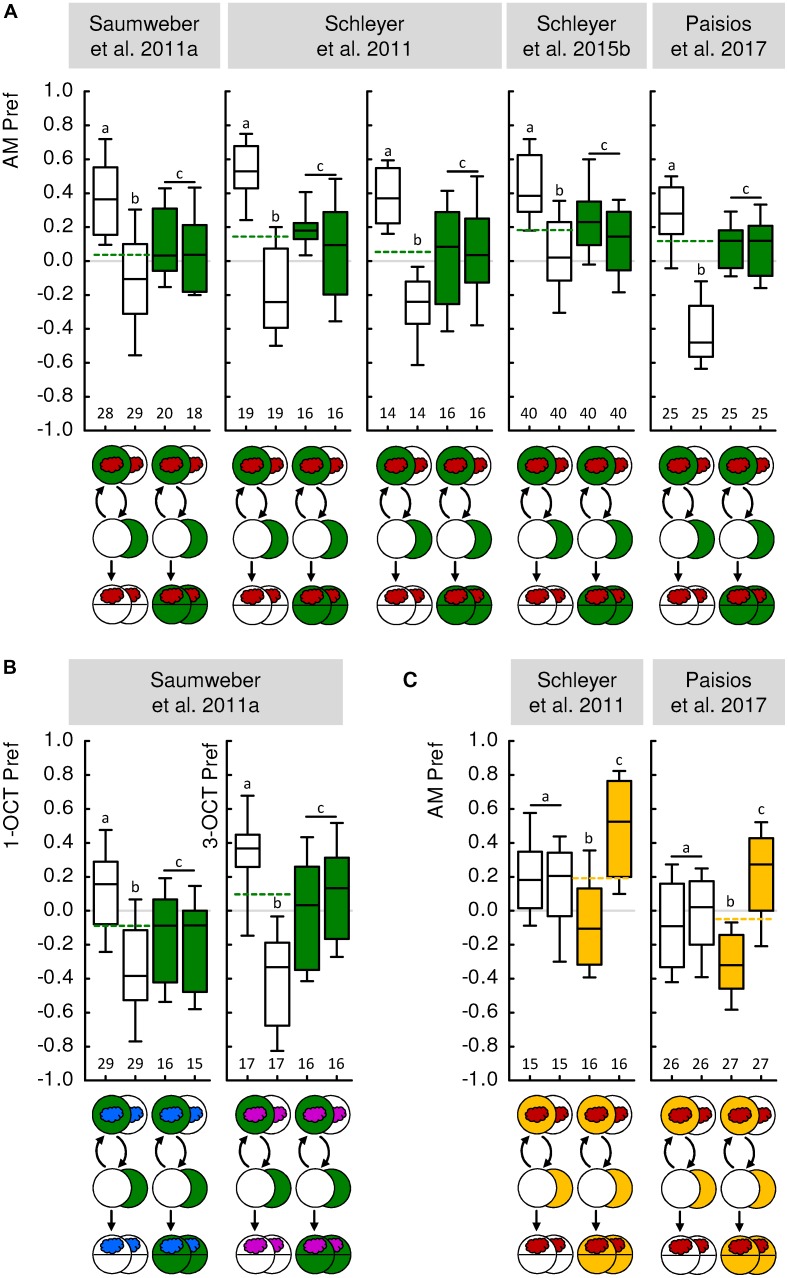
Paired and unpaired memory in larval *Drosophila*. **(A)** In five independent experiments from four different previously published studies, paired and unpaired memory was demonstrated using *n*-amyl acetate as the odor (AM, red cloud) and fructose as the reward (green-filled circles). In a Petri-dish assay, odor and reward were presented paired such that the odor was presented while the animals were on a reward-containing Petri dish; the animals were then transferred to an empty Petri dish without odor or reward (white-filled circle). In an independent group of animals, odor and reward were presented unpaired, in consecutive trials. When tested for their preference for the odor (AM Pref), the larvae preferred the odor more after paired than after unpaired training (white-filled box plots). When tested on a reward-containing Petri dish, however, the larvae displayed an intermediate level of odor preference that was the same regardless of the training regimen (green-filled box plots). This can therefore serve as a baseline for odor preference in animals that have established, but do not behaviorally express, associative odor memory (stippled line). Such a procedure reveals that memory through reward-paired training increases, whereas memory through reward-unpaired training decreases odor preference relative to this baseline. **(B)** Same as **(A)**, but using either 1-octanol (1-OCT, blue cloud) or 3-octanol (3-OCT, purple cloud) as odors. In two independent, previously published experiments, paired and unpaired memory was demonstrated using *n*-amyl acetate as the odor (AM, red cloud) and quinine as punishment (yellow-filled circles). Only when tested for their odor preference on a punishment-containing Petri dish did the larvae avoid the odor more after paired than after unpaired training (yellow-filled box plots). When tested in the absence of punishment, the larvae displayed an intermediate level of odor preference that was the same regardless of the training regimen (white-filled box plots, yellow stippled line). Thus, punishment-paired training decreases, whereas punishment-unpaired training increases odor preference relative to the baseline odor preference shown in the absence of the punishment. Data were taken from the publications indicated above each experiment. For more details on experimental parameters and the methods used, see **Table 1** as well as the Methods sections of the indicated papers. Box plots indicate the median as the middle line and the 25/75% and 10/90% quantiles as box boundaries and whiskers, respectively. Sample sizes are displayed below each box-plot. In all cases, the preference values were statistically indistinguishable between the training regimens when animals were tested under baseline conditions [Mann–Whitney *U*-tests (MW), *P* > 0.05 corrected according to Bonferroni–Holm within each experiment], indicated by a common letter and a vertical bar above the box plots. The stippled line indicates the median of the pooled preference data under baseline conditions. The preferences after paired and after unpaired training differed from each other, as well as from baseline (MW, *P* < 0.05 corrected according to Bonferroni–Holm within each experiment), as indicated by different letters above the box plots. For detailed statistical results see **Supplementary Table S1**.

**FIGURE 2:**
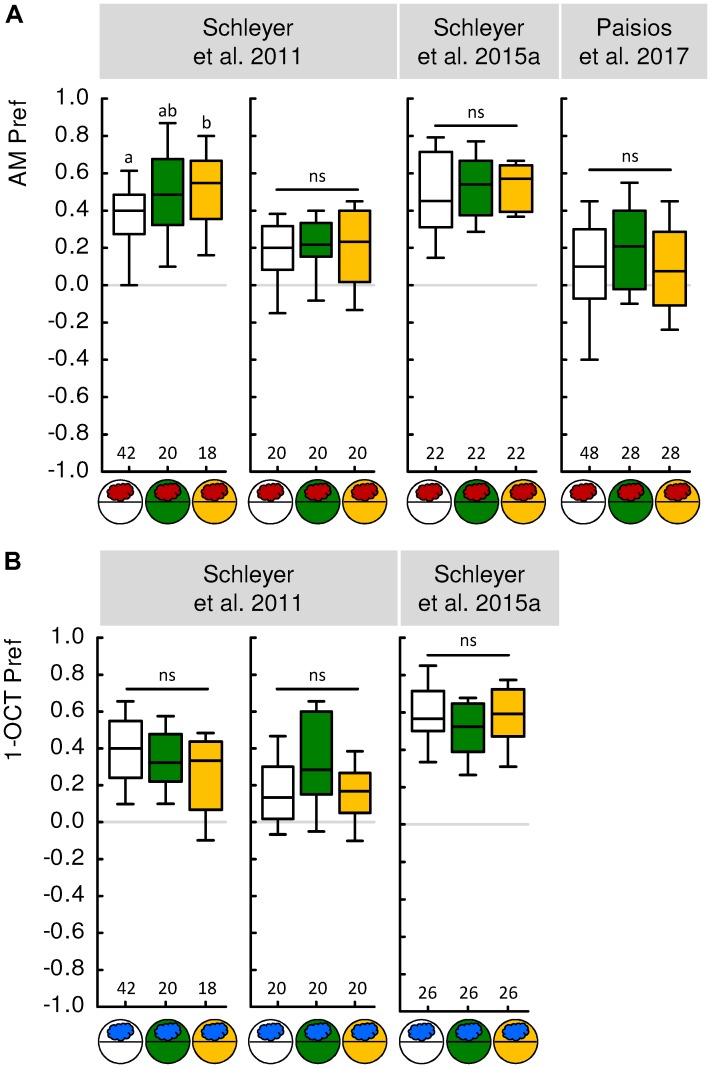
Innate odor preference is not affected by reinforcer presence. **(A)** Experimentally naive larvae were tested for their preference for *n*-amyl acetate as odor (AM, red cloud) either on a tasteless substrate (white circles), on fructose (green circles), or on quinine (yellow circles). The results from four independent experiments and three different studies, using slightly different parameters such as odor concentration or Petri dish size, are displayed. Innate AM preference was largely the same regardless of the presence of a reinforcer. This contrasts with the massive influence of reinforcer presence on learned AM preference (**Figure 1**). **(B)** Same as **(A)**, but using 1-octanol (1-OCT, blue cloud) as odor. Data were taken from the publications indicated above each experiment. For more details on experimental parameters and the methods used, see **Table 2** as well as the Methods sections of the indicated papers. Sample sizes are displayed below each box plot. Statistically indistinguishable odor preferences are indicated by “n” (Kruskal-Walles test, *P* > 0.05). Small letters above box plots indicate significant differences between odor preferences (MW, *P* < 0.05 corrected according to Bonferroni-Holm within each experiment). For detailed statistical results see **Supplementary Table S2**. Other details as in **Figure 1**.

For both larval and adult *Drosophila*, one-odor ‘absolute’ conditioning paradigms are available (larvae: Saumweber et al., 2011a; adults: Niewalda et al., 2011). For example, larvae are repeatedly transferred between two types of Petri dish featuring substrates that are supplemented, or not, with a taste reward (**Figure 1**). An odor A is presented together with a sugar-containing substrate; a tasteless substrate is then presented without an odor (A+/blank, paired training). These animals can learn that the reward can be found where the odor is (see also **Supplementary Figure S1B**). Importantly, a second group of larvae is trained unpaired, i.e., the odor and the reward are present on different dishes (A/+, unpaired training). These animals can learn that the reward can be found where the odor is not (see also **Supplementary Figure S1C**). After typically three such training cycles, the animals are transferred to a test Petri dish where their preference for A is assessed. This usually reveals a higher preference for A after paired than after unpaired training (**Figure 1A**, the two left-most box-plots of each panel). This difference in preference between paired-trained and unpaired-trained animals indicates how much the contingency between odor and reward matters for the larvae’s odor preference, and can thus serve as a measure of associative memory. However, is this due to associative memory in the paired-trained group, associative memory in the unpaired-trained group, or both? The observation that the larvae approach or avoid the odor after a given training procedure is not in itself an argument in this respect, because odors are not neutral to experimentally naive larvae, but support moderate levels of attraction (**Figure 2**) (Cobb, 1999; Saumweber et al., 2011a). This being so, can the behavior of experimentally naive larvae be used as a baseline against which to measure effects of paired and unpaired training? We argue that such a comparison would be misguided. Relative to both paired- and unpaired-trained animals, experimentally naive animals lack not only the target associative experiences, but also the experience of handling, of exposure to the odor, and of exposure to the reward – experiences that can evidently all affect odor preference (larvae: Boyle and Cobb, 2005; Michels et al., 2005; Colomb et al., 2007; Saumweber et al., 2011b; adults: Préat, 1998; Sadanandappa et al., 2013; Niewalda et al., 2015; Hattori et al., 2017). The same applies to measures of odor preference after handling-only (lacking the target associative experience and exposure to the odor and the reward), after odor-only exposure (lacking the associative experience and reward-exposure), or after reward-only exposure (lacking the associative experience and odor-exposure). In other words, using any of the above-mentioned procedures to establish a baseline odor preference can lead analyses of associative memory astray. A better option would be to expose animals to both odor and reward with a truly randomized temporal relationship between them (Rescorla, 1966, 1967, 1968). In such a randomized procedure, the probability of the reinforcer occurring would be the same in the presence as in the absence of the odor, and the odor would thus not provide any information about the reinforcer. It has been shown that animals may nevertheless associatively learn in such a procedure, depending on the specific parameters of the experiment and the exact sequence of events (discussed in Rescorla, 1972; Papini and Bitterman, 1990). Still, if that appropriate parameters are used, the truly randomized procedure can provide a baseline against which to measure the effects of paired and unpaired training. Indeed, it has been successfully used in the case of fear conditioning in the rat, for example, as will be discussed in the Section “Unpaired-Memory in Rodents?”. Even so, a randomized procedure is only feasible if training consists of sufficiently many trials (Rescorla, 1972), and not in cases when only a handful of trials are used, as in the paradigms discussed for *Drosophila* and honeybees.

A second strategy is not to try to prevent the formation of associative memory, but to prevent its behavioral expression. How can this be done? Fortunately, the behavioral expression of associative memory in larvae has been found to depend on the circumstances of testing (Hendel et al., 2005; Schleyer et al., 2011, 2015a,b; Paisios et al., 2017). Specifically, the behavioral expression of odor-reward memories both after paired and after unpaired training is fully suppressed when the test is carried out in the presence of the reward. That is, if the test is conducted in the presence of the reward, the larvae behave the same toward the odor regardless of whether they have undergone paired or unpaired training. This can be understood as adaptive if one views conditioned behavior as a search for reward that is obsolete if the sought-for reward is already present (Gerber and Hendel, 2006; Schleyer et al., 2011, 2015a,b; see also Craig, 1918). Significantly, animals tested in this way have experienced the same amount of handling, odor exposure and sugar exposure, and will have even formed the same associative memories as animals trained the same but tested in the absence of the reward. Thus, none of these aspects of experience can account for differences in test behavior in the presence versus in the absence of the reward. What the presence of the reward during the test does is to prevent the behavioral expression of associative memory, i.e., to abolish the difference in odor preference between paired-trained and unpaired-trained animals (**Figure 1**, green box plots). This is an effect specific to learned behavior, as olfactory behavior in experimentally naive larvae is not likewise affected (**Figure 2**). The equal level of odor preference in paired-trained and unpaired-trained animals in the presence of the reward can thus be used as a baseline, reflecting olfactory behavior specifically cleared of associative memories. The following Section “Evidence for Unpaired-Memory in Larval *Drosophila*” discusses what a re-analysis of previously published experiments using such a baseline approach can reveal about the memories formed though paired and unpaired training of odor and taste reinforcement.

### Evidence for Unpaired-Memory in Larval *Drosophila*

**FIGURE 3:**
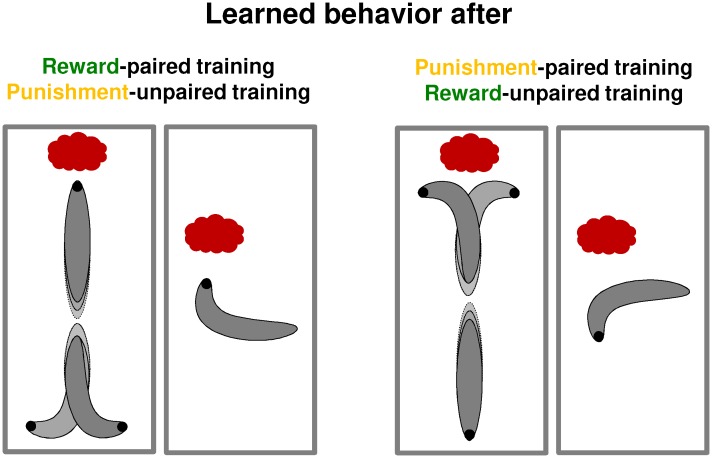
Microbehavior after paired and unpaired training. In summary of the studies by Schleyer et al. (2015b) and Paisios et al. (2017), this schematic overview shows the microbehavioral changes after paired and unpaired training. These changes depend on the valence of memory, not on the type of reinforcer. That is, larvae display the same microbehavior after reward-paired and punishment-unpaired learning on the one hand, whereas the opposite effects are observed after both punishment-paired and reward-unpaired training on the other hand. Left: after reward-paired and after punishment-unpaired training, larvae turn less while moving toward the odor source, turn more while moving away from it, and bias the direction of their turns more toward the odor source than animals under baseline conditions. As a result, they approach the odor. Right: after punishment-paired or reward-unpaired training, the same behavioral aspects are modulated, yet with opposite sign, leading to odor avoidance.

The first experiment including such a baseline condition was reported by Saumweber et al. (2011a) with *n*-amyl acetate as the odor and fructose as the reward. In this and the following analyses, we pooled the data for paired-trained and unpaired-trained animals tested under baseline conditions (e.g., **Figure 1A**, green box plots and stippled line), and compared them to animals that were paired-trained or unpaired-trained and tested under non-baseline conditions (e.g., **Figure 1A**, blank box plots), using pairwise statistical tests (for details, see the “Materials and Methods” section in the **Supplementary Presentation S1**). Associative memory after paired or unpaired training would manifest itself as a difference between the respective group and the baseline. Indeed, paired odor-reward training increased odor preference compared to baseline, whereas unpaired training decreased odor preference compared to baseline (**Figure 1A**). This result has been reproduced four times in three follow-up studies (**Figure 1A**) (Schleyer et al., 2011, 2015b; Paisios et al., 2017) and confirmed using two further odors (**Figure 1B**) (Saumweber et al., 2011a). Interestingly, it was shown that the resulting increase and decrease in odor preference, respectively, come about by opposite modulations of the microbehavioral tendencies that underlie chemotaxis. After paired training, larvae turn less while moving toward the odor source, turn more while moving away from it, and bias the direction of their turns more toward the odor source than animals under baseline conditions do; after unpaired training, the larvae modulate the very same parameters of their locomotion, yet in the opposite way (**Figure 3**) (Schleyer et al., 2015b; Paisios et al., 2017). Together these analyses show that *Drosophila* larvae do indeed acquire associative memories during paired training and during unpaired training, and that these memories are opposite in valence and in the ‘sign’ of microbehavioral modulation. What about the aversive domain?

*Drosophila* larvae can be conditioned to associate odors with taste punishment such as highly concentrated salt, or quinine (Gerber and Hendel, 2006; Niewalda et al., 2008; El-Keredy et al., 2012). Importantly, the associative memories established by such training are behaviorally expressed only in the presence but not in the absence of the taste punishment (Hendel et al., 2005; Gerber and Hendel, 2006; Schleyer et al., 2011, 2015a; Paisios et al., 2017). This can be understood if conditioned behavior after punishment training is viewed as an escape from the punishment, which is obsolete in the absence of anything to escape from (Gerber and Hendel, 2006; Schleyer et al., 2011, 2015a; see also Craig, 1918). Given that innate olfactory behavior in experimentally naive animals is not likewise affected by the presence of punishing tastants (**Figure 2**), this makes it possible to measure odor preference after paired or unpaired punishment training, and to compare the levels of preference against baseline – which in this case is determined by testing the animals in the absence of the punishment. It turned out that after paired odor-punishment training, larvae prefer the odor less than at baseline, whereas after unpaired punishment training they prefer the odor more than at baseline (**Figure 1C**) (Schleyer et al., 2011; Paisios et al., 2017). In other words, after paired training the larvae seek to escape from the punishment by heading where the odor is not, whereas after unpaired training they seek to escape from the punishment by heading where the odor is. In terms of microbehavior, the comparison to baseline revealed that turn rate and turn direction are modulated in opposite ways after paired versus unpaired punishment-training (Paisios et al., 2017). Specifically, memories after reward-paired and punishment-unpaired training affect these aspects of locomotion in the same way, whereas opposite modulations were observed after both reward-unpaired and punishment-paired training (**Figure 3**) (Paisios et al., 2017).

These results show two points of conceptual relevance. Firstly, the way in which microbehavior is affected is determined by memory valence, not by the used reinforcer: for example, when heading toward the odor source turns are suppressed both by reward-paired and by punishment-unpaired memory (**Figure 3**). This is adaptive because in both cases it keeps the larvae on target (i.e., on track toward the odor). Secondly, the way in which the presence of the reinforcer during the test affects the behavioral expression of memory is determined in turn by the used reinforcer, not by memory valence (**Supplementary Figure S2**): for example, the presence of the reward suppresses the behavioral expression of reward-memory both after reward-paired and after reward-unpaired training, although these two types of training establish memory of opposite valence. This is adaptive because in both cases learned behavior is about obtaining the desired outcome (i.e., the reward).

Unpaired learning may explain otherwise enigmatic observations, for example that mutations of learning-related genes affect odor preference after paired and unpaired training with opposite sign (Michels et al., 2011; Saumweber et al., 2011b; Kleber et al., 2016). Likewise, training with higher concentrations of a reward or higher intensity of punishment has opposite effects on odor preference after paired versus unpaired training (El-Keredy et al., 2012; Schleyer et al., 2015a).

### Unpaired-Memory After Differential Conditioning of Larval *Drosophila*

Traditionally, most studies of Pavlovian conditioning in *Drosophila* employ differential, two-odor conditioning (adults: Quinn et al., 1974; Tempel et al., 1983; Tully and Quinn, 1985; larvae: Aceves-Pina and Quinn, 1979; Scherer et al., 2003; Neuser et al., 2005). These procedures are identical to the conditioning paradigms described above, except that an additional odor B is introduced. The larvae receive one odor paired with reinforcement whereas another odor is presented alone (i.e., unpaired from reinforcement) (A+/B training). Subsequently, they are tested for their choice between A and B. If after such A+/B training the animals prefer A over B, this is usually interpreted as caused by the A+ association. Arguably, however, such preference for A over B may be driven by two associative behavioral tendencies: the animals may be attracted to A because it signals where the reward is, and/or they may be repelled by B because it signals where the reward is not (see also Rescorla, 1969 and references therein). Thus, in this type of paradigm it is impossible to disentangle the contribution of either of these two processes. This is required, however, to fully appreciate how experience with reinforcement shapes behavior.

**FIGURE 4:**
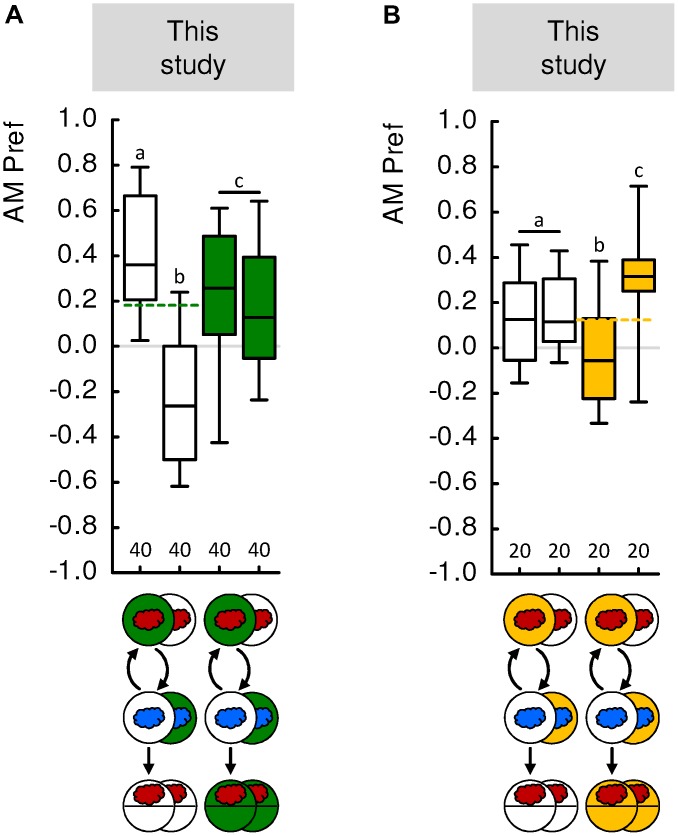
Paired and unpaired memory upon differential conditioning. **(A)** In this previously unpublished experiment, larvae were trained in the differential, two-odor version of the learning experiment. The experiments followed established procedures (Gerber et al., 2013; Michels et al., 2017). In brief, we used 2 mol/L fructose as the reward, and *n*-amyl acetate diluted 1:20 in paraffin (AM, red cloud) as well as undiluted 1-octanol (1-OCT, blue cloud) as odors. Larvae were trained such that AM and 1-OCT were presented in consecutive trials for 2.5 min each. In this differential conditioning paradigm, for one group of animals AM was always presented on a reward-containing Petri dish and 1-OCT on a tasteless Petri dish (left-most box plot). A second group was trained reciprocally, such that 1-OCT was always paired with reward (second box-plot from the left). After three such training cycles, the larvae were transferred to a test Petri dish, where their odor preference for AM was determined. When tested in the absence of the reward, the larvae prefer AM more after it was paired with the reward than after it was not paired with the reward (white-filled box plots). When tested in presence of the reward, the animals display an intermediate level of odor preference that is the same regardless of the training regimen (green-filled box plots). Thus, when AM was trained paired with the reward during differential conditioning, the larvae learned that it signals where the reward is, whereas when AM was trained unpaired with the reward during differential conditioning, the larvae learned that it signals where the reward is not. **(B)** As in **(A)**, but using 5 mmol/L quinine hemisulfate as punishment. When tested in the presence of the punishment, the larvae avoid AM more after it was paired with the punishment during differential conditioning than after unpaired training (yellow-filled box plots). When tested in the absence of the punishment, the larvae display an intermediate level of odor preference that is the same regardless of the training regimen (white-filled box plots). Thus, when AM was trained paired with the punishment during differential conditioning, the larvae learned that it signals where the punishment is, whereas when AM was trained unpaired with the punishment during differential conditioning, the larvae learned that it signals where the punishment is not. Sample sizes are displayed below each box plot. For detailed statistical results see **Supplementary Table S3**. For detailed methods, see the “Materials and Methods” section in the **Supplementary Presentation S1**. Other details as in **Figure 1**.

To address this problem, we modified the differential, two-odor conditioning paradigm (see “Materials and Methods” section in the **Supplementary Presentation S1**; see also Saumweber et al., 2011a; for adults: Barth et al., 2014). In these previously unpublished experiments, we first trained larvae ‘normally’ such that in one group of animals odor A was paired with a fructose reward but odor B was presented alone (A+/B), whereas in an independent group of animals, contingencies were reversed (A/B+). However, we then did not test the animals for their choice between odor A and B, but rather determined their absolute preference for odor A versus blank. This allowed us to assess the preference for odor A after it had been presented, during differential conditioning, either paired or unpaired with the reward. These preferences for odor A were then compared to baseline, i.e., to the preference for odor A after the same type of training but tested in the presence of the reward. This revealed that the preference for odor A is above baseline if, during differential conditioning, it has been the paired-trained odor, and below baseline if it has been the unpaired-trained odor (**Figure 4A**). We conclude that during differential conditioning, too, larvae learn both about the reward-paired and about the reward-unpaired odor. The same principle, with reversed sign, applies in the aversive domain as well (**Figure 4B**).

## Unpaired-Memory in Adult Flies and Honeybees?

The ‘baseline approach’ discussed above has so far only been used in larval *Drosophila*. Is there evidence from other kinds of experimental approach warranting the conclusion that unpaired learning takes place in adult flies, or honeybees?

### Adult Flies

To the best of our knowledge, no unequivocal, direct evidence is available from adult *Drosophila* that unpaired learning takes place. However, for a number of observations unpaired memory is a parsimonious explanation.

Using absolute conditioning paradigms with odor and electric shock as a punishment, preference scores have in some studies been reported separately for paired-trained and unpaired-trained flies. In the study by Niewalda et al. (2011), for example, avoidance was found for four different odors after paired odor-punishment training, whereas unpaired training resulted in odor attraction (see also Yarali et al., 2009; Barth et al., 2014; König et al., 2017). The latter result is suggestive of unpaired memory because the odors in question, and in fact odors in general, are innately repulsive to adult *Drosophila* in the type of setup used (de Belle and Heisenberg, 1994; Préat, 1998; Acevedo et al., 2007; Knapek et al., 2010; Niewalda et al., 2015). Indeed, when the effect of odor concentration on memory performance was evaluated, increasing the odor concentration increased the odor attraction observed after unpaired training (Niewalda et al., 2011) – whereas in experimentally naive flies increasing the odor concentration makes the odors more aversive (Tully and Quinn, 1985). Still, it remains possible that such odor attraction reflects the effects of handling, or of shock-exposure, or of odor-exposure that are part of the training experience. Because training-like odor-exposure and training-like shock-exposure typically only decrease aversion without converting it into attraction (Préat, 1998; Knapek et al., 2010), however, unpaired-memory seems to be the more likely explanation for these effects.

Similarly suggestive data were recently reported by Cohn et al. (2015) from an isolated-brain preparation. The authors used stimulation of olfactory interneurons of the so-called mushroom body (‘odor’) paired or unpaired with activation of dopaminergic reward neurons (DANs) innervating them. They then measured the physiological effect of such training at the level of the output neurons of the mushroom body (MBONs) using Ca^2+^-imaging. ‘Olfactory’ activation paired with DAN activity depressed subsequent MBON activity in response to ‘odor,’ whereas upon unpaired presentations MBON activity was potentiated. Arguably, and with the same caveats in mind as discussed in the preceding paragraph, these opposite modulations of MBON activity could reflect paired and unpaired memory.

### Honeybees

Honeybees are a widely used study case for learning and memory, both under natural and under laboratory conditions (Giurfa, 2007; Menzel, 2012). In the present context, studies using Pavlovian reward learning of the proboscis extension response (PER) are particularly relevant. Individual honeybees are harnessed such that they can freely move their antennae and mouthparts, including their proboscis. When their antennae are touched with a sucrose solution as a reward, the bees reflexively extend their proboscis and lick the sucrose; few if any such PERs are typically observed when odors are presented to experimentally naive animals. During PER conditioning, an odor A is presented shortly before a reward (A+; in Pavlovian terminology the CS and US, respectively). After such paired training, increased levels of PER are observed in response to the odor alone. Obviously, without modification this paradigm cannot detect memories of opposite valence after unpaired training (A/+): as spontaneous PER rates are low they remain low after unpaired training. In other words, there is no ‘negative’ PER that could reveal unpaired-memory. One modification allowing such unpaired-memory to be detected is called retardation of acquisition. In such a two-phase paradigm the bees of two independent experimental groups first receive either paired or unpaired reward-training. In a second training phase, the bees of both groups receive paired training (paired-paired group: A+ training followed by A+ training; unpaired-paired group: A/+ followed by A+). As first reported by Bitterman et al. (1983), during the second training phase the bees in the unpaired-paired group respond less to A than those in the paired-paired group. Given that presentations of odor-alone or of reward-alone during the initial training phase do not have such an effect, this shows that unpaired training establishes an associative memory opposite in valence to paired training in the PER paradigm.

Data from two-odor, differential PER conditioning are consistent with, but are not in themselves conclusive evidence for, unpaired learning. In the course of an extended differential conditioning phase (A+, B, A+, B, A+, B, etc.), levels of PER toward B are initially elevated, arguably because of generalization from the first A+ training trial. As training progresses, however, the response levels to B decrease (Bitterman et al., 1983; Komischke et al., 2002; Boitard et al., 2015; see also Tedjakumala and Giurfa, 2013 for similar results in the aversive domain). This could be due either to a loss of generalized memory (the end-state being no memory for B) or to unpaired learning (the end-state being unpaired-memory for B).

The physiological data regarding the effects of unpaired training in the honeybee are complex. The PE1 neuron, an MBON from the peduncle of the mushroom body, has been shown to decrease its activity to an odor that was previously trained paired with reward (Mauelshagen, 1993; Okada et al., 2007). Regarding an unpaired-trained odor, mild increases or decreases in PE1 activity can be observed depending on trial number and time after odor onset (Mauelshagen, 1993; Okada et al., 2007). Also in other MBONs, in the antennal lobe and in the octopaminergic rewarding VUM_mx1_ neuron, altered responses to unpaired odors have been observed (Hammer, 1993; Faber et al., 1999; Strube-Bloss et al., 2011). These effects are typically small compared to the effects of reward-paired odors, and in no case have proper baseline levels of activity been determined. Therefore, alternative interpretations of these physiological data, such as non-associative learning or extinction learning, which are well documented in honeybees (Braun and Bicker, 1992; Hammer et al., 1994; Menzel et al., 1999; Eisenhardt and Menzel, 2007; Eisenhardt, 2014), remain tenable.

## Unpaired-Memory in Rodents?

In the Section “How to Determine Baseline Odor Preferences in Larval *Drosophila*” we described a strategy in studying larval *Drosophila* that provides a baseline against which the associative effects of paired and unpaired training can be assessed - a strategy that prevents associative memories from being behaviorally expressed under baseline conditions. As mentioned, historically it was a different strategy that was applied to determine baseline behavior, namely preventing the formation of associative memories (Rescorla, 1966, 1967, 1968). In the following, we focus on fear conditioning in rodents as one study case for which that strategy has been used.

In laboratory rats or mice, non-reinforcement of a stimulus was often assumed to be mnemonically neutral and was thus used as a control in Pavlovian conditioning. For example, in differential fear conditioning a stimulus such as a tone A (serving as CS) is repeatedly paired with a punishing foot-shock reinforcement (+) (serving as US) (Ciocchi et al., 2010; Lange et al., 2014; Wigestrand et al., 2017). Intended as a control, a stimulus B, which can be a tone of another frequency, is presented in the absence of punishment. In most studies, B is presented before the beginning of the A training period (B, B, B, …, A+, A+, A+, etc.) (e.g., Lange et al., 2014). In fewer studies, B is presented during the A training period but unpaired from punishment (A+, B, A+, B, A+, B, etc.) (Wigestrand et al., 2017). Either way, a retention test is carried out, typically a day later. This test involves presenting A and B, in separate trials, and in a novel context. If the animals have learned the predictive relationship between A and shock, they should show freezing behavior upon the presentation of A, i.e., a species-specific defensive behavior consisting of a crouched body position and a cessation of all body movements except breathing. Provided that A and B are sufficiently distinct (Laxmi et al., 2003), freezing is observed upon presenting A but not upon presenting B, and not upon presenting a novel, not previously presented stimulus C. Does this mean that no learning about B has taken place? Not necessarily. This is because freezing is a monovalent measure, just like the PER in honeybees. In a novel context the animals hardly freeze, and thus only increases in freezing caused by negatively valenced memories can be measured. Positively valenced, unpaired memory for B, if it existed, would go unnoticed, since the animals cannot freeze less than not at all. To detect unpaired-learning, therefore, either the retardation-of-acquisition approach discussed above can be used (Pollak et al., 2010), or a bivalent measure is needed that allows positively and negatively valenced memories to be detected by modulations of the same behavioral read-out, with opposite sign. As will be discussed in the following section, up- and down-regulation of moderate levels of contextual freezing or of the startle response can provide such bivalent measures in rodents. In both cases the idea is to induce an affective state that can then either be potentiated by negatively valenced memory or attenuated by positively valenced memory.

**Table 1:** Synopsis of parameters varying across experiments on reinforcement-unpaired learning in *Drosophila* larvae.

Odor (dilution)	Reinforcer (concentration)	Training trial duration	Petri dish diameter	Comments	Published in	Shown in this paper
*n*-amyl acetate (1:20)	Fructose (2 mol/L)	5 min	9 cm	First demonstration (reward)	Saumweber et al., 2011a, Figure 6	**Figure 1A**
*n*-amyl acetate (1:50)	Fructose (0.2 mol/L)	5 min	9 cm		Schleyer et al., 2011, **Supplementary Figure S4**	**Figure 1A**
*n*-amyl acetate (1:50)	Fructose (2 mol/L)	5 min	9 cm		Schleyer et al., 2011, **Supplementary Figure S5**	**Figure 1A**
*n*-amyl acetate (1:50)	Fructose (0.2 mol/L)	5 min	15 cm	Includes analysis of microbehavior	Schleyer et al., 2015b, Figures 1, 3, 4	**Figure 1A**
*n*-amyl acetate (1:50)	Fructose (2 mol/L)	2.5 min	15 cm	Includes analysis of microbehavior	Paisios et al., 2017, Figures 2, 4	**Figure 1A**
1-octanol (1:1)	Fructose (2 mol/L)	5 min	9 cm		Saumweber et al., 2011a, Figure 6	**Figure 1B**
3-octanol (1:2)	Fructose (2 mol/L)	5 min	9 cm		Saumweber et al., 2011a, Figure 6	**Figure 1B**
*n*-amyl acetate (1:50)	Quinine (5 mmol/L)	5 min	9 cm	First demonstration (punishment)	Schleyer et al., 2011, **Supplementary Figure S5**	**Figure 1C**
*n*-amyl acetate (1:50)	Quinine (5 mmol/L)	5 min	15 cm	Includes analysis of microbehavior	Paisios et al., 2017, Figures 2, 4	**Figure 1C**
*n*-amyl acetate (1:20), 1-octanol (1:1)	Fructose (2 mol/L)	2.5 min	9 cm	Differential training, absolute odor preference	This study	**Figure 4A**
*n*-amyl acetate (1:20), 1-octanol (1:1)	Quinine (5 mmol/L)	2.5 min	9 cm	Differential training, absolute odor preference	This study	**Figure 4B**

*For the evidence for reinforcement-unpaired learning in Drosophila larvae mentioned in this study, the critical experimental parameters and site of publication of the original studies are presented.*

### Bivalent Measures of Valence in Rodents

One suggested approach to measuring positively valenced memory after unpaired training takes advantage of the contextual learning capabilities of rodents (Ostroff et al., 2010; Pollak et al., 2010; Kong et al., 2014). In these experiments, the test takes place in a context in which the animals have previously received foot-shock punishment. Within such a punishment-predicting context, the animals show the freezing behavior described above. To serve as a bivalent measure, it is important that the levels of freezing displayed by the animals should be moderate because this prevents floor or ceiling effects. If in this situation a stimulus A is presented that has itself been unpaired-trained with foot shock, the context-induced freezing is attenuated. By contrast, context-induced freezing is potentiated if A has been paired with shock. One interpretation is that through unpaired training the animals have learned that whenever A is present, punishment will not occur (*a.k.a.* safety learning). However, as discussed in Section “How to Determine Baseline Odor Preferences in Larval *Drosophila*,” a firm conclusion would require a proper baseline measure of freezing to disentangle whether the difference in freezing between paired-trained and unpaired-trained conditions results from either one of these two types of training, or from both.

**Table 2:** Synopsis of parameters varying across experiments on innate odor preference.

Odor (dilution)	Reinforcer (concentration)	Petri dish diameter	Published in	Shown in this paper
*n*-amyl acetate (1:50)	Fructose (2 mol/L), Quinine (5 mmol/L)	9 cm	Schleyer et al., 2011, Figure 4	**Figure 2A**
*n*-amyl acetate (1:10000)	Fructose (2 mol/L), Quinine (5 mmol/L)	9 cm	Schleyer et al., 2011, Figure 4	**Figure 2A**
*n*-amyl acetate (1:50)	Fructose (2 mol/L), Quinine (5 mmol/L)	9 cm	Schleyer et al., 2015a, Figure 3	**Figure 2A**
*n*-amyl acetate (1:50)	Fructose (2 mol/L), Quinine (5 mmol/L)	15 cm	Paisios et al., 2017, **Supplementary Figure S2**	**Figure 2A**
1-octanol (1:1)	Fructose (2 mol/L), Quinine (5 mmol/L)	9 cm	Schleyer et al., 2011, Figure 4	**Figure 2B**
1-octanol (1:10000)	Fructose (2 mol/L), Quinine (5 mmol/L)	9 cm	Schleyer et al., 2011, Figure 4	**Figure 2B**
1-octanol (1:1)	Fructose (2 mol/L), Quinine (5 mmol/L)	9 cm	Schleyer et al., 2015a, Figure 3	**Figure 2B**

*For the experiments on the effects of reinforcement presence on innate odor preference in Drosophila larvae mentioned in this study, the critical experimental parameters and site of publication of the original studies are presented.*

A second approach takes advantage of the startle response, which can be elicited by a sudden, loud noise (*a.k.a.* the startle probe). This response consists of a short-latency contraction of all body muscles and can be measured by motion-sensitive devices (reviews include Koch, 1999; Fendt and Koch, 2013). To use the startle response as a read-out for a learning experiment, the animals are first trained with pairings of a stimulus A with foot-shock punishment. For the test, the startle probe is delivered either in the presence of A or in its absence. If the animals have learned the predictive relationship between A and punishment, the startle magnitude is higher in the presence than in the absence of A (*a.k.a* fear-potentiated startle, Davis et al., 1993; Fendt and Fanselow, 1999). This difference, i.e., startle in the presence of A minus startle in the absence of A, is quantified as the Startle Difference Score. Importantly for the present discussion, previously rewarded stimuli exert the opposite effect, i.e., startle is attenuated in their presence (Schmid et al., 1995). Thus, modulations of the startle response can be used as a bivalent measure of memory: positively valenced memories decrease startle, whereas negatively valenced memories increase it. Does this allow unpaired-memory to be revealed? Indeed, when A is presented unpaired from punishment during training, an attenuation of the startle response is observed in the test (Falls and Davis, 1994; Richardson and Fan, 2002). In this case too, one interpretation is that the animals have learned that whenever A is present, punishment will not occur (*a.k.a.* safety learning). However, a firm conclusion would again require a proper baseline against which to measure startle after unpaired training.

Thus, although both approaches offer bivalent measures of valence, both approaches as such also fall short of providing a proper baseline against which the effects of paired versus unpaired memory can be measured. How can such a baseline be determined?

### Evidence for Unpaired-Memory in Rats

To determine the baseline response to a stimulus A free of associative effects of either paired or unpaired training, a procedure is needed in which no predictive relationship exists between A and punishment. To this end, Rescorla (1967) introduced the ‘truly random’ procedure. The idea is that A and punishment occur in a randomized temporal relationship. This means that A and punishment can also, by chance, occur together. If properly implemented, after truly random training A does not predict anything (for a more detailed discussion see Rescorla, 1972; Papini and Bitterman, 1990), whereas after unpaired training A predicts the non-occurrence of punishment (which is therefore often designated ‘explicitly unpaired’ training). We note that despite this critical difference in experimental outcome, the Methods sections of surprisingly many publications do not specifically state whether an unpaired or a truly random procedure was used. This would be important, however, in order to properly interpret the results from experiments that use these procedures. In the present paper, we use ‘unpaired’ in the sense of explicitly unpaired throughout.

**FIGURE 5:**
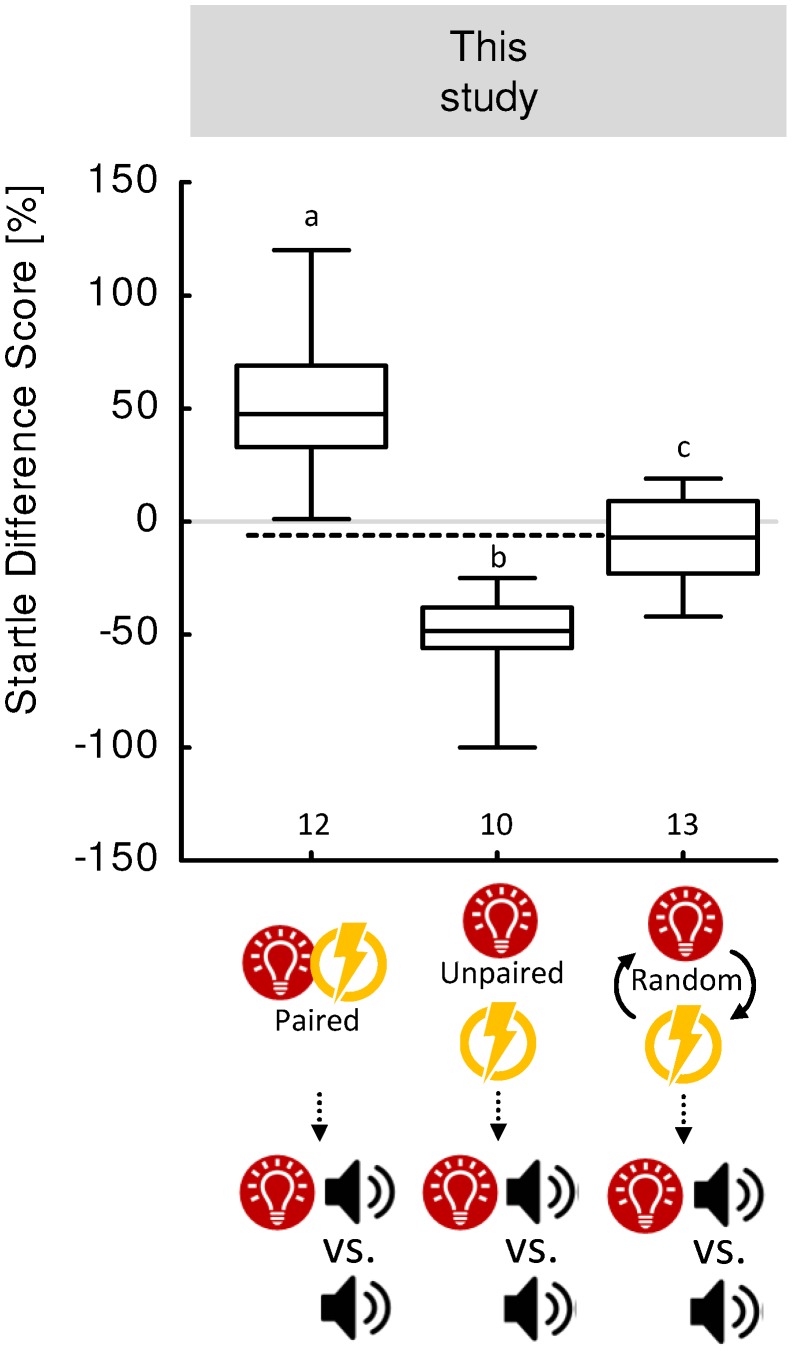
Paired and unpaired memory in rats. In this previously unpublished experiment replicating a study by Andreatta et al. (2012), independent groups of rats were submitted to paired training of a light stimulus and punishment **(left)**, to unpaired training **(middle)**, or to a truly random procedure **(right)**. Specifically, a light stimulus (red circle) and a mild foot-shock (yellow flash) were presented 15 times. For paired training, the light stimulus immediately preceded the shock (intertrial interval, ITI: 90–150 s); for unpaired training, the light stimulus and the shock were temporally separated from each other by at least 12 s; and in the random procedure, the light stimulus and the shock were randomly presented. One day later (vertical dotted arrow), the effects of the light stimulus on the startle response were measured. Startle probes (noise from a loudspeaker) were presented in the presence or absence of the light stimulus. Plotted are the Startle Difference Scores, i.e., the mean startle magnitude in the presence of the light minus mean startle magnitude in the absence of the light. Sample sizes are displayed below each box plot. For detailed statistical results see **Supplementary Table S4**. For detailed methods, see the “Materials and Methods” section in the **Supplementary Presentation S1**. Other details as in **Figure 1**.

Using the truly random procedure, startle has turned out to be the same in the presence and in the absence of A, i.e., the Startle Difference Scores are zero (Davis and Astrachan, 1978; Hitchcock and Davis, 1987; Risbrough et al., 2003; Hsu et al., 2012). After unpaired training, by contrast, animals startle less in the presence of A, i.e., the Startle Difference Scores are negative (Falls and Davis, 1994; Richardson and Fan, 2002). However, neither of these studies directly compared the outcome of the two types of training. Indeed, to the best of our knowledge the first study to do so was Andreatta et al. (2012). Their data confirmed that after unpaired training startle is attenuated in the presence of A, whereas following a truly random procedure this is not the case. Critically, the Startle Difference Scores are lower after unpaired training than after the truly random procedure. As these data thus provided the first and, to our knowledge, so far the only direct evidence for unpaired-memory in rats, we here include a hitherto unpublished replication of the experiment in question, with slightly modified parameters (**Figure 5**) (see also the “Materials and Methods” section in the **Supplementary Presentation S1**). Rats were submitted to 15 presentations of a light stimulus A (5 s duration) and foot-shock punishment (0.5 s duration, 0.4 mA) with intertrial-intervals ranging between 90 and 150 s. Different groups of rats underwent one of three training conditions: (1) for one group stimulus A preceded the shock (Paired group); (2) one group received unpaired presentations of A and shock, such that the inter-stimulus-interval was never shorter than 12 s (Unpaired group); and (3) one group underwent the truly random procedure (Random group, i.e., baseline). Confirming Andreatta et al. (2012), startle was potentiated by the presence of A in the Paired group, attenuated in the Unpaired group, and unaffected in the Random group. Critically, the Startle Difference Scores were more negative in the Unpaired than in the Random group.

To summarize, from experiments using startle modulation as a bivalent behavioral read-out and the truly random procedure to determine baseline behavior, we conclude that paired and unpaired training establish oppositely valenced associative memories in rats.

## Concluding Discussion and Outlook

The evidence presented from larval and adult *Drosophila*, honeybees, and rats confirms a general principle of classical experimental psychology: that animals learn through both paired and unpaired presentations of a stimulus A with reinforcement, and that the resulting associative memories are opposite in valence. This warns against using the unpairing of A with a reward or punishment as a control for the effects of associative learning. Indeed, unpaired presentations of A and reinforcement are not a safe procedure in controlling for associative learning effects – because such a procedure can in itself establish associative memory for A as a signal that a reward or punishment will not occur.

Importantly, as we show here, this applies not only to ‘absolute,’ non-differential conditioning, but to differential conditioning as well: when stimulus A is presented paired with reinforcement and, in the same experimental subjects, another stimulus B is presented unpaired from reinforcement, larval *Drosophila* associatively learn about both stimuli – with opposite valence. Arguably, the behavior after any differential conditioning experiment might thus be a result of either or both of two types of learning process that need to be disentangled in order to fully understand the results.

Despite being established knowledge in classical psychology, the principle of opposite memories through paired and unpaired training is often neglected in neuroscience and genetics. As a consequence, the underlying mechanisms, be it on the circuit and neuronal level or the genetic and molecular level, are largely unknown. We have here presented two behavioral approaches to studying unpaired learning in two different model organisms. These approaches can now be adapted in order to unravel its underlying the mechanisms underlying unpaired learning and memory. Research in insects can play a crucial role in this endeavor. The *Drosophila* larva in particular has demonstrated its potential for in-depth analyses of the genetic and neuronal mechanisms of reinforcement learning (Schroll et al., 2006; Michels et al., 2011; Rohwedder et al., 2016; Widmann et al., 2016; Eichler et al., 2017; Saumweber et al., 2018).

A full appreciation of unpaired learning would prompt a re-evaluation of the conclusions drawn from experiments comparing the effects of paired training with unpaired-control conditions, whether in differential or in non-differential ‘absolute’ conditioning paradigms. Although these approaches are useful to describe the outcome of associative learning in general, they cannot disentangle the effects of paired and unpaired training. If knocking-down a gene or neuronal population is found to reduce memory scores in such a task, it remains uncertain whether this gene or neuronal population is important for paired memory, or unpaired memory, or both. Likewise, if different physiological responses are elicited by a paired-trained and an unpaired-trained stimulus, it remains to be established whether effects of paired training, of unpaired training, or both are responsible for the difference. In any event, future research across species will be required to reveal whether unpaired learning as a behavioral principle is based on common mechanistic principles. If this were found to be the case, such research might help us to understand how our own behavior comes about.

## Ethics Statement

All experiments were carried out in accordance with international guidelines for the use of animals in experiments (2010/63/EU). All experiments with rats were approved by the local ethical committee (Landesverwaltungsamt Sachsen-Anhalt, Az. 42502-2-1309 UniMD).

## Data Availability

The data for all presented behavioral experiments can be found in the **Supplementary Data Sheet S1**.

## Author Contributions

MS, MF, and BG wrote the manuscript. SS and MS performed and analyzed the experiments displayed in **Figure 4**.

## Conflict of Interest Statement

The authors declare that the research was conducted in the absence of any commercial or financial relationships that could be construed as a potential conflict of interest.
