# Supplementary material for: Associative Learning of Stimuli Paired and Unpaired With Reinforcement: Evaluating Evidence From Maggots, Flies, Bees, and Rats
Source: Front Psychol. 2018 Aug 24;9:1494. doi: 10.3389/fpsyg.2018.01494 (PMC6117914; doi:10.3389/fpsyg.2018.01494)
Supplement: Supplementary file 1 [file Presentation_1.pdf]

Associative learning of stimuli paired and unpaired with reinforcement: Evaluating evidence from maggots, flies, bees and rats

Michael Schleyer[1]*, Markus Fendt[2,3], Sarah Schuller[1], Bertram Gerber[1,3,4]

[1] Leibniz Institute for Neurobiology (LIN), Department Genetics of Learning and Memory, Magdeburg, Germany
[2] Otto von Guericke University Magdeburg, Institute for Pharmacology and Toxicology, Magdeburg, Germany
[3] Center of Behavioral Brain Science (CBBS), Magdeburg, Germany
[4] Otto von Guericke University Magdeburg, Institute for Biology, Behavior Genetics, Magdeburg, Germany

*Corresponding author:
michael.schleyer@lin-magdeburg.de

Supplementary information

Material and Methods

This paper discusses both previously published and unpublished experiments, all of which follow established methods (Andreatta et al., 2012; Gerber et al., 2013; Michels et al., 2017). Figures 1-2 present previously published data; Figs. 4-5 present new, hitherto unpublished experiments. In this section, we first report the statistical approaches applied to all data, and then briefly summarize how the previously unpublished experiments were performed. The raw data underlying all figures can be found in the Supplementary Datasheet 1. Key parameters varying across the *Drosophila* learning experiments are summarized in Table 1 and Table 2.

Statistical Analyses

Two-tailed non-parametric tests were used (statistical assumptions for these tests were met throughout). Values were compared across multiple groups with Kruskal-Wallis tests (KW test). For subsequent pair-wise comparisons, Mann-Whitney U-tests (MW test) were used. To test whether values of a given group differ from chance, i.e. from zero, we used one-sample sign tests (OSS test). When multiple comparisons were performed within one analysis, a Bonferroni-Holm correction was applied to keep the experiment-wide error rate below 5 % (Holm, 1979). For KW and MW tests, we used Statistica 11.0 (Statsoft), for OSS tests R 3.4.0 (R Core Team, 2017). In all learning experiments on *Drosophila* (Fig. 1-3), the preference values were statistically indistinguishable between the training procedures when animals were tested under baseline conditions (MW tests, $P > 0.05$ corrected according to Bonferroni-Holm within each experiment), indicated by a common letter and a vertical bar above the box plots. Therefore, we pooled these data and compared the pooled data with the preferences after paired and after unpaired training when tested in non-baseline conditions (MW tests). The median of the pooled baseline data is displayed as a stippled line. Experimenters were blind to the testing conditions. We present our data as box plots which represent the median as the middle line and 25%/75% and 10%/90% as box boundaries and

whiskers, respectively. Outliers are not displayed. Sample sizes are displayed below each box-plot. In order to keep the main text and the figure legends concise, we report the results of statistical tests in Tables S1-S4.

Experiments with *Drosophila* Larvae

The methods employed for the previously unpublished experiments (Fig. 4) largely followed established procedures (Gerber et al., 2013; Michels et al., 2017), for the most part also matching those employed in the previous publications from which the data in Figure 1 were taken; for a synopsis of key parameters varying across learning experiments, see Table 1. In brief, we used third-instar feeding-stage larvae from the Canton-Special wild-type strain, aged 5 days after egg laying. Flies were maintained on standard medium, in mass culture at 25 °C, 60-70 % relative humidity and a 12/12 hour light/dark cycle. Before each experiment, we removed a spoonful of food medium from a food vial, collected about 20 larvae, briefly rinsed them in distilled water and started the experiment.

For the experiments we used Petri dishes of 90 mm inner diameter (Sarstedt, Nümbrecht, Germany) filled with 1 % agarose (electrophoresis grade; Roth, Karlsruhe, Germany). As reinforcers fructose (FRU; 2 mol/L; CAS: 57-48-7; Roth, Karlsruhe, Germany) or quinine (QUI; 5 mmol/L; CAS: 207671-44-1; Sigma-Aldrich, Steinheim, Germany) were used. As odors, we used *n*-amyl acetate (AM; CAS: 628-63-7; Sigma-Aldrich, Steinheim, Germany), diluted 1:50 in paraffin oil (Merck, Darmstadt, Germany) and undiluted 1-octanol (OCT; CAS: 111-87-5; Merck, Darmstadt, Germany).

Prior to experiments, odor containers were prepared: 10 µl of odor substance was filled into custom-made Teflon containers (5 mm inner diameter with a lid perforated with seven 0.5-mm diameter holes). Before the experiment started, Petri dishes were covered with modified lids perforated in the center by 15 holes of 1 mm diameter to improve aeration.

For training, approximately 20 larvae were placed in the middle of a FRU-containing dish with two odor containers on opposite sides, both filled with AM. After 2.5 min, the larvae were transferred onto an agarose-only dish with two containers filled with OCT, where they also spent 2.5 min. Three such AM+/OCT training cycles were performed, in each case using fresh dishes. In repetitions of the experiment, in half of the cases training started with a FRU-containing dish (AM+/OCT) and in the other half with an agarose-only dish (OCT/AM+). For each group of larvae trained AM+/OCT (or OCT/AM+, respectively), a second group was trained reciprocally, i.e.: AM/OCT+ (or OCT+/AM, respectively).

Following training, the larvae were transferred to a test Petri dish that did or did not contain FRU and were given the choice between AM on one side, and an empty odor container (EM) on the other side of the dish. After three minutes the larvae were counted and a preference score was calculated as:

(1) $\text{AM Pref} = (\#_{AM} - \#_{EM}) / \#_{Total}$

In this equation, # indicates the number of larvae on the respective half of the dish. Thus, AM Pref values are constrained between 1 and -1, with positive values indicating approach towards, and negative values indicating aversion of AM.

With quinine as the reinforcer, the experiments were performed and the Preferences were calculated in an analogous way.

Experiments with Rats

Adult Sprague-Dawley rats were reared under standard conditions (i.e. group housing with *ad libitum* food and water in a temperature- and humidity-controlled vivarium) and, when 8-10 weeks of age, were admitted to experiments that used an 8-box startle system (SR-LAB,

San Diego Instruments, San Diego, USA) equipped with floor grids (for applying the foot shock US), light bulbs (for applying the light CS) and loudspeakers (for presenting the startle probe). Rats were submitted to three different conditioning protocols. In these protocols, a 5-s light stimulus and a 0.5-mA foot shock were presented 15 times each with variable intertrial-intervals (90-150 s from shock to shock). In the 'Paired' training condition, the light stimulus preceded and co-terminated with the foot shock. In the 'Unpaired' group, the light stimulus was presented randomly, but was never less than 12 s before or after the foot shock. In the 'Random' group, the light stimulus was presented randomly, i.e. also very shortly before or after the foot shock. One day later, a startle test was performed in which the startle probe (a noise of 96 dB SPL and 40 ms duration) was presented every 30 s, either in the presence or the absence of the light stimulus. The startle amplitude (SA) was measured by a motion sensor underneath the device. The percent difference between these two testing conditions was calculated as:

(2) Startle Difference Score = (SA in presence – SA in absence) * 100 / SA in absence

Thus, positive scores reflect an increased startle in the presence of the light stimulus, which is indicative of negative valence, whereas negative scores reflect a decreased startle in the presence of the light stimulus, indicative of positive valence.

References

Andreatta, M., Fendt, M., Muhlberger, A., Wieser, M. J., Imobersteg, S., Yarali, A., Gerber, B., and Pauli, P. (2012). Onset and offset of aversive events establish distinct memories requiring fear and reward networks. *Learn Mem*, 19, 518-26

Gerber, B., Biernacki, R., and Thum, J. (2013). Odor-taste learning assays in *Drosophila* larvae. *Cold Spring Harb Protoc*, 2013, pdb.prot071639.

Holm, S. (1979). A simple sequentially rejective multiple test procedure. *Scand J Statist*, 6, 65-70

Michels, B., Saumweber, T., Biernacki, R., Thum, J., Glasgow, R. D. V., Schleyer, M., Chen, Y. C., Eschbach, C., Stocker, R. F., Toshima, N., Tanimura, T., Louis, M., Arias-Gil, G., Marescotti, M., Benfenati, F., and Gerber, B. (2017). Pavlovian conditioning of larval *Drosophila*: an illustrated, multilingual, hands-on manual for odor-taste associative learning in maggots. *Front Behav Neurosci*, 11, 45

R Core Team (2017). R: A language and environment for statistical computing. *R Foundation for Statistical Computing.*

Source Data

Supplementary Datasheet 1: Behavioral source data underlying all experiments displayed in the article. Each page of the Excel file contains the data of one main figure.

Supplementary Figures

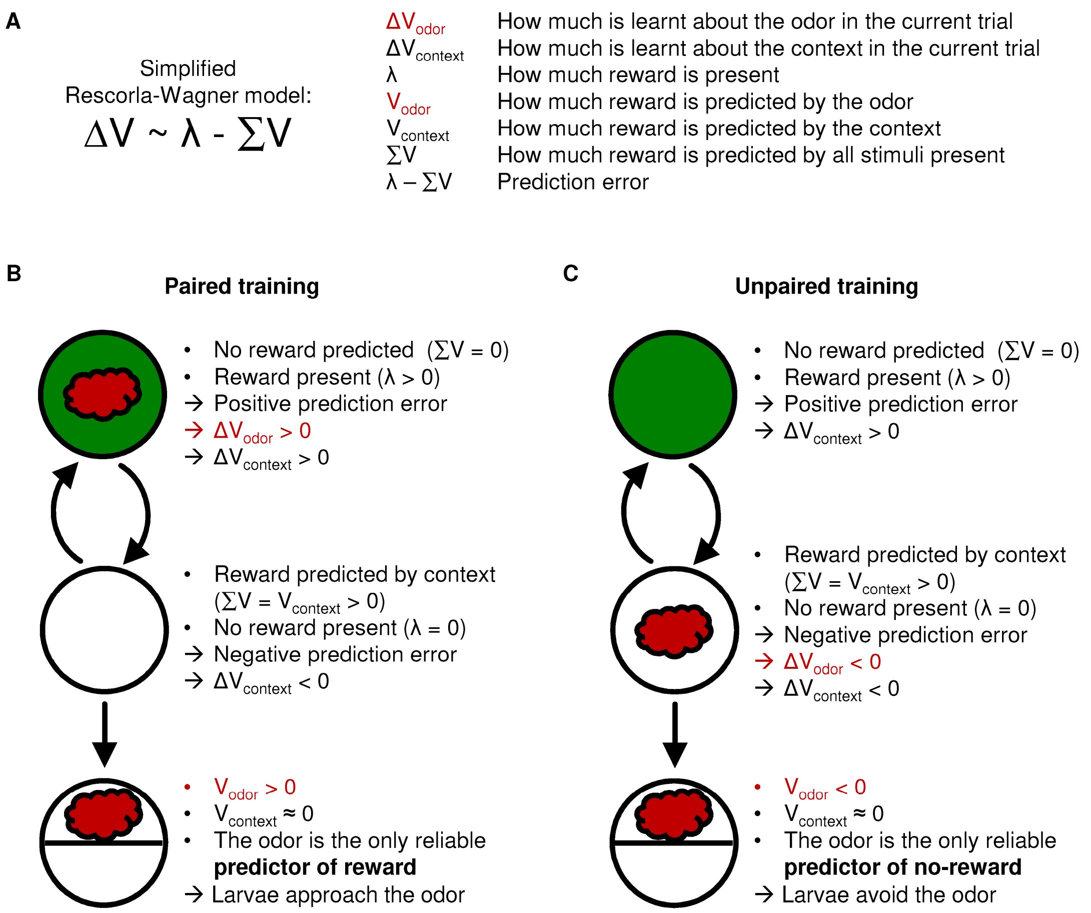

Figure S1: Paired and unpaired learning in larval *Drosophila*, according to the Rescorla-Wagner model

(A) According to the Rescorla-Wagner model, the amount (and sign) of learning during a learning trial (ΔV) is proportional to the prediction error, comprising the current reward strength (λ) minus the reward strength predicted by all present stimuli ($\sum V$).

(B) At the beginning of an experiment of paired odor-reward training, no reward is predicted as the whole experimental context as well as the odor is novel to the larvae. The presence of a reward (green fill) is thus unpredicted and results in a positive prediction error. This prediction error is associated with all stimuli present, i.e. the odor (red cloud) and the contextual cues of the Petri dish (e.g. light, physical properties of agarose surface). If the larvae are then transferred to a second Petri dish, the context will predict a reward – but no reward is actually present. The resulting negative prediction error is associated with the context but not with the odor (because the odor is not present). Across repetitions of this cycle, the context is alternately associated with a positive and a negative prediction error. The odor, however, is only associated with positive prediction errors. Thus, at the moment of the test, the odor (and not the context) reliably predicts the reward and is therefore approached by the larvae.

(C) At the beginning of unpaired training too, reward is present but not predicted. Importantly, the resulting positive prediction error is associated with the context but not the odor (because the odor is not present). After transferring the larvae to the second, odor-containing Petri dish, the context predicts a reward, but no reward is present. The resulting negative prediction error is associated with the context, as well as the odor. Across repetitions of this cycle, the context is alternately associated with a positive and a negative prediction error. The odor, however, is only associated with negative prediction errors. Thus, at the moment of the test, the odor reliably predicts no reward and is therefore avoided by the larvae.

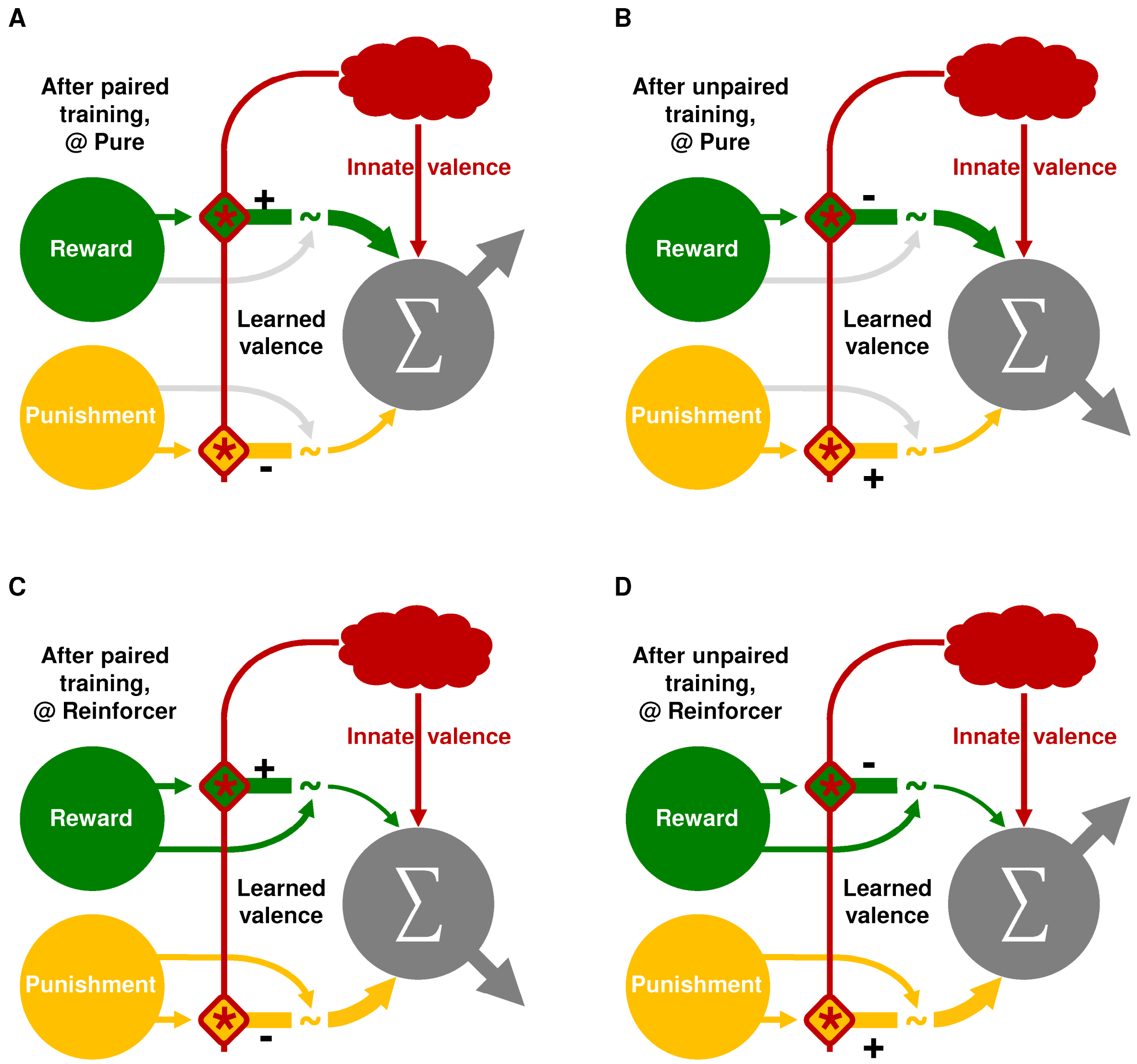

Figure S2: Schematic of how innate and learned valence jointly determine behavioral output
Rewards (green) can be trained paired **(A, C)** or unpaired **(B, D)** with the odor (red cloud) to establish an associative memory (indicated by *). Reward-paired training leads to a positive learned-valence bias, whereas reward-unpaired training leads to a negative learned-valence bias (indicated by + and -, respectively). Likewise, punishments (yellow) can be trained paired or unpaired with the odor, causing a negative learned-valence bias after punishment-paired training and a positive learned-valence bias after punishment-unpaired training. Learned-valence biases are summed with the innate valence of the odor to a common valence signal that determines olfactory behavior. Significantly, reward-related memories enter the summation with the innate valence only in the absence of a reward **(A, B)**, but are prevented from affecting behavior in the presence of the reward **(C, D)** (indicated by ~, and reduced line strength). This suppression is independent of the valence of the memory (i.e. takes place for both paired- and unpaired reward-memory), and specifically affects reward-related and not punishment-related memories. Punishment-related memories, in turn, are prevented from affecting behavior in the absence of punishment **(A, B)**, and only enter the summation in the presence of it **(C, D)**. The proposed gating processes (~) are suggested to occur upstream of the summation point indicated by ∑, and do not affect innate valence signaling. Please note that physiologically, paired-memories likely correspond to a depression of synaptic strength and that learned valence is likely computed by combining pathways with approach- and avoidance-promoting effects.

Supplementary tables

Table S1: Statistical results for previously published learning experiments on *Drosophila* larvae

Displayed in this study, label of original publication	Test	Results	Displayed in this study, label of original publication	Test	Results
Fig. 1A, labeled as from Saumweber et al. 2011	KW	**df = 3, H = 28.5, $P = 0$**	Fig. 1B, labeled as from Saumweber et al. 2011, left display	KW	**df = 3, H = 25.8, $P = 0$**
	MW (paired vs. unpaired)	**U = 105.5, $P = 0$**		MW (paired vs. unpaired)	**U = 109.5, $P = 0$**
	MW (baseline)	U = 165.5, $P = 0.68$		MW (baseline)	U = 114.5, $P = 0.84$
	MW (paired vs. baseline)	**U = 227.5 $P = 0$**		MW (paired vs. baseline)	**U = 228.5, $P = 0.0011$**
	MW (unpaired vs baseline)	**U = 368, $P = 0.021$**		MW (unpaired vs baseline)	**U = 301.5, $P = 0.029$**
	OSS (paired)	**$P = 0$**		OSS (paired)	$P = 0.26$
	OSS (unpaired)	$P = 0.18$		OSS (unpaired)	**$P = 0.0001$**
	OSS (baseline)	$P = 0.50$		OSS (baseline)	$P = 0.043$
Fig. 1A, labeled as from Schleyer et al. 2011, left display	KW	**df = 3, H = 40.1, $P = 0$**	Fig. 1B, labeled as from Saumweber et al. 2011, right display	KW	**df = 3, H = 27.3, $P = 0$**
	MW (paired vs. unpaired)	**U = 0, $P = 0$**		MW (paired vs. unpaired)	**U = 15, $P = 0$**
	MW (baseline)	U = 128, $P = 1$		MW (baseline)	U = 97, $P = 0.25$
	MW (paired vs. baseline)	**U = 79.5, $P = 0.0006$**		MW (paired vs. baseline)	**U = 139.5, $P = 0.0056$**
	MW (unpaired vs baseline)	**U = 91.5, $P = 0.0016$**		MW (unpaired vs baseline)	**U = 92, $P = 0.0002$**
	OSS (paired)	**$P = 0$**		OSS (paired)	**$P = 0.013$**
	OSS (unpaired)	$P = 0.064$		OSS (unpaired)	**$P = 0.0003$**
	OSS (baseline)	**$P = 0.0070$**		OSS (baseline)	$P = 0.28$
Fig. 1A, labeled as from Schleyer et al. 2011, right display	KW	**df = 3, H = 26.2, $P = 0$**	Fig. 1C, labeled as from Schleyer et al. 2011	KW	**df = 3, H = 19.7, $P = 0.0002$**
	MW (paired vs. unpaired)	**U = 5.5, $P = 0$**		MW (baseline)	U = 119, $P = 0.75$
	MW (baseline)	U = 103, $P = 0.35$		MW (paired vs. unpaired)	**U = 25, $P = 0.0001$**
	MW (paired vs. baseline)	**U = 56.5, $P = 0$**		MW (paired vs. baseline)	**U = 119, $P = 0.0028$**
	MW (unpaired vs baseline)	**U = 117, $P = 0.0003$**		MW (unpaired vs baseline)	**U = 133.5, $P = 0.0076$**
	OSS (paired)	**$P = 0.0001$**		OSS (baseline)	**$P = 0.011$**
	OSS (unpaired)	**$P = 0.0001$**		OSS (paired)	**$P = 0.005$**
	OSS (baseline)	$P = 0.28$		OSS (unpaired)	$P = 0.21$
Fig. 1A, labeled as from Schleyer et al. 2015b	KW	**df = 3, H = 47.7, $P = 0$**	Fig. 1B, labeled as from Paisios et al. 2017	KW	**df = 3, H = 38.4, $P = 0$**
	MW (paired vs. unpaired)	**U = 181, $P = 0$**		MW (baseline)	U = 290, $P = 0.38$
	MW (baseline)	U = 585, $P = 0.059$		MW (paired vs. unpaired)	**U = 45.5, $P = 0$**
	MW (paired vs. baseline)	**U = 698.5, $P = 0$**		MW (paired vs. baseline)	**U = 315.5, $P = 0$**
	MW (unpaired vs baseline)	**U = 1014.5, $P = 0.0011$**		MW (unpaired vs baseline)	**U = 338.5, $P = 0.0002$**
	OSS (paired)	**$P = 0$**		OSS (baseline)	$P = 0.89$
	OSS (unpaired)	$P = 0.29$		OSS (paired)	**$P = 0$**
	OSS (baseline)	**$P = 0$**		OSS (unpaired)	**$P = 0.0094$**
Fig. 1A, labeled as from Paisios et al. 2017	KW	**df = 3, H = 56.5, $P = 0$**			
	MW (paired vs. unpaired)	**U = 5.5, $P = 0$**			
	MW (baseline)	U = 293.5, $P = 0.72$			
	MW (paired vs. baseline)	**U = 333.5, $P = 0.0011$**			
	MW (unpaired vs baseline)	**U = 45, $P = 0$**			
	OSS (paired)	**$P = 0.0001$**			
	OSS (unpaired)	**$P = 0$**			
	OSS (baseline)	**$P = 0.021$**			

All results from the statistical tests performed on the data from the previously published learning experiments on Drosophila *larvae that are presented in this study (Fig. 1) are documented. Significant results (corrected according to Bonferroni-Holm) are in bold.*

Table S2: Statistical results for innate preference experiments on *Drosophila* larvae

Displayed in this study, label of original publication	Test	Results	Displayed in this study, label of original publication	Test	Results
Fig. 2A, labeled as from Schleyer et al. 2011, left display	KW	**df = 2, H = 6.7, P = 0.034**	Fig. 2A, labeled as from Schleyer et al. 2015a	KW	df = 2, H = 0.89, P = 0.64
	MW (PUR vs FRU)	U = 293.5, P = 0.058		OSS (pooled)	**P = 0**
	MW (PUR vs QUI)	**U = 236.5, P = 0.023**	Fig. 2A, labeled as from Paisios et al. 2017	KW	df = 2, H = 3.4, P = 0.19
	MW (FRU vs QUI)	U = 175, P = 0.89		OSS (pooled)	**P = 0.0022**
	OSS (PUR)	**P = 0**	Fig. 2B, labeled as from Schleyer et al. 2011, left display	KW	df = 2, H = 4.6, P = 0.10
	OSS (FRU)	**P = 0**		OSS (pooled)	**P = 0**
	OSS (QUI)	**P = 0.001**	Fig. 2B, labeled as from Schleyer et al. 2011, right display	KW	df = 2, H = 5.2, P = 0.076
				OSS (pooled)	**P = 0**
Fig. 2A, labeled as from Schleyer et al. 2011, right display	KW	df = 2, H = 0.16, P = 0.92	Fig. 2B, labeled as from Schleyer et al. 2015b	KW	df = 2, H = 2.8, P = 0.25
	OSS (pooled)	**P = 0**		OSS (pooled)	**P = 0**

All results from the statistical tests performed on the data from the previously published experiments on innate odor preference that are presented in this study (Fig. 2) are documented. Significant results (corrected according to Bonferroni-Holm) are in bold.

Table S3: Statistical results for previously unpublished learning experiments on *Drosophila* larvae

Displayed in this study	Test	Results	Displayed in this study	Test	Results
Fig. 4A	KW	**df = 3, H = 53, P = 0**	Fig. 4B	KW	**df = 3, H = 13.9, P = 0.0029**
	MW (paired vs. unpaired)	**U = 128, P = 0**		MW (baseline)	U = 178.5, P = 0.57
	MW (baseline)	U = 673.5, P = 0.23		MW (paired vs. unpaired)	**U = 90.5, P = 0.0019**
	MW (paired vs. baseline)	**U = 1089, P = 0.0045**		MW (paired vs. baseline)	**U = 241.5, P = 0.013**
	MW (unpaired vs baseline)	**U = 560.5, P = 0**		MW (unpaired vs baseline)	**U = 259.5, P = 0.015**
	OSS (paired)	**P = 0**		OSS (baseline)	**P = 0.0026**
	OSS (unpaired)	**P = 0.0005**		OSS (paired)	P = 0.82
	OSS (baseline)	**P = 0**		OSS (unpaired)	**P = 0.0015**

All results from the statistical tests performed on the data from the previously unpublished learning experiments on Drosophila *larvae that are presented in this study (Fig. 4) are documented. Significant results (corrected according to Bonferroni-Holm) are in bold.*

Table S4: Statistical results for previously unpublished learning experiments on rats

Displayed in this study	Test	Results
Fig. 5	KW	**df = 3, H = 23.5, P = 0**
	MW (paired vs. unpaired)	**U = 0, P = 0**
	MW (paired vs. baseline)	**U = 19, P = 0.0015**
	MW (unpaired vs baseline)	**U = 11.5, P = 0.0010**
	OSS (paired)	**P = 0.0063**
	OSS (unpaired)	**P = 0.0020**
	OSS (baseline)	P = 0.58

All results from the statistical tests performed on the data from the previously unpublished learning experiments on rats that are presented in this study (Fig. 5) are documented. Significant results (corrected according to Bonferroni-Holm) are in bold.
